# Evaluation of an Exercise Field Test Using Heart Rate Monitors to Assess Cardiorespiratory Fitness and Heart Rate Recovery in an Asymptomatic Population

**DOI:** 10.1371/journal.pone.0097704

**Published:** 2014-05-21

**Authors:** Crystal L. Coolbaugh, Ivan B. Anderson, Machelle D. Wilson, David A. Hawkins, Ezra A. Amsterdam

**Affiliations:** 1 Biomedical Engineering Graduate Group, University of California Davis, Davis, California, United States of America; 2 Division of Cardiovascular Medicine, Department of Internal Medicine, University of California Davis, Sacramento, California, United States of America; 3 Division of Biostatistics, Department of Public Health Sciences, University of California Davis, Davis, California, United States of America; 4 Department of Neurobiology, Physiology, and Behavior, University of California Davis, Davis, California, United States of America; Scuola Superiore Sant'Anna, Italy

## Abstract

**Purpose:**

Measures of cardiorespiratory fitness (CRF) and heart rate recovery (HRR) can improve risk stratification for cardiovascular disease, but these measurements are rarely made in asymptomatic individuals due to cost. An exercise field test (EFT) to assess CRF and HRR would be an inexpensive method for cardiovascular disease risk assessment in large populations. This study assessed 1) the predictive accuracy of a 12-minute run/walk EFT for estimating CRF (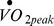) and 2) the accuracy of HRR measured after an EFT using a heart rate monitor (HRM) in an asymptomatic population.

**Methods:**

Fifty subjects (48% women) ages 18–45 years completed a symptom-limited exercise tolerance test (ETT) (Bruce protocol) and an EFT on separate days. During the ETT, 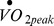 was measured by a metabolic cart, and heart rate was measured continuously by a HRM and a metabolic cart.

**Results:**

EFT distance and sex independently predicted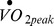. The average absolute difference between observed and predicted 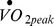 was 0.26±3.27 ml·kg^−1^·min^−1^ for our model compared to 7.55±3.64 ml·kg^−1^·min^−1^ for the Cooper model. HRM HRR data were equivalent to respective metabolic cart values during the ETT. HRR at 1 minute post-exercise during ETT compared to the EFT had a moderate correlation (r = 0.75, p<0.001).

**Conclusion:**

A more accurate model to estimate CRF from a 12-minute run/walk EFT was developed, and HRR can be measured using a HRM in an asymptomatic population outside of clinical settings.

## Introduction

Risk assessment is the foundation for primary prevention of future cardiovascular disease, yet attempts to evaluate the large population of asymptomatic individuals are limited [1], [2]. Clinical exercise tolerance tests (ETTs) to quantitatively assess cardiorespiratory fitness (CRF) and heart rate recovery (HRR) post-exercise can improve risk stratification [3]–[5], but the expense of testing large populations [6], [7] and low primary care utilization rates in young adults [8] limits clinical efforts for early risk detection. Exercise field tests (EFTs) completed while wearing commercial heart rate monitors (HRMs) may provide an inexpensive alternative for risk assessment in large populations, but improved CRF prediction models and validation of HRR measures are needed. A regression model, developed by Cooper in 1968 [7], has been widely used to estimate CRF (peak oxygen uptake (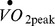)) for a 12-minute run/walk EFT, but the predictive accuracy of the model is dependent on the population being tested. In the original study, 115 male, military officers with an average age of 22 years (range 17 to 52) and a moderate range of 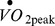 values (31–59 ml·kg^−1^·min^−1^) and 12-minute run/walk distances (1770–3218 m) were tested [9]. Application of the Cooper model in similar populations of moderately fit men has yielded accurate estimates of CRF [10]–[13]; however, CRF predictions are often underestimated when the model has been used for women or subjects with lower fitness levels [11], [14], [15]. Development of a model based on a diverse range of fitness levels that includes subject characteristics (e.g. age, sex, and body composition) [16], [17], therefore, may improve CRF estimates. In addition, use of a HRM during the EFT affords continuous and accurate measurement of heart rate in response to exercise and during recovery. The validity of HRR measured by a HRM outside of clinical settings, however, has not been examined. The purposes of this study were to 1) develop a CRF (peak oxygen uptake (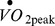)) prediction model applicable to both men and women with a greater fitness range than the Cooper model [9] and 2) evaluate the accuracy of HRR during a 12-minute run/walk EFT in an asymptomatic, low-risk population.

## Methods

### Ethics Statement

The University of California, Davis Institutional Review Board approved the protocol, and all subjects gave written informed consent.

### Participants

Participants, age 18–45 years were screened and were excluded if a moderate or high risk for a cardiovascular event during exercise was identified [18]. This population was considered because it includes a portion of the people we hope to be able to screen in large numbers to identify those warranting physician consultation and possible additional risk stratification. A total of 26 men and 24 women were enrolled (Table 1). No specific criteria were used ensure a diversity in fitness levels of the subject population, but efforts were made to enroll subjects with an even distribution of activity levels ranging from sedentary to very active. Sample size was based on the desired width of confidence intervals for parameters in the regression model to predict 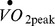. In this approach, the probability, ⋎, that the confidence interval (at level α) will be no wider than twice a desired half-width *w* was specified [19]. We selected ⋎ = 0.8, α = 0.05, and *w* = 0.3. We assumed the highest correlation between predictor variables did not exceed 0.7, and the final R^2^ of the model would be approximately 0.7, a conservative estimate compared to previous correlations [9]. A minimum sample size of 45 subjects was required to achieve these specifications for any given parameter estimate. Additional subjects were recruited to account for subject drop-out.

**Table 1:** Demographic, resting, and exercise parameters of participants.

	Men	Women	p
Age (years)	28.9±7.6	31.0±7.4	= .3335
Height (m)	1.78±0.62	1.63±0.65	<.0001
Mass (kg)	78.0±8.8	59.4±8.2	<.0001
BMI (kg·m^−2^)	24.5±2.1	22.2±2.4	= .0008
Resting			
Heart Rate (bpm)	68.4±13.3	71.3±12.9	= .4429
Systolic Blood Pressure (mmHg)	119.0±9.7	110.5±8.7	= .0020
Diastolic Blood Pressure (mmHg)	74.2±8.9	72.4±11.8	= .5398
Exercise Tolerance Test			
Exercise Time (min)	13.5±1.9	11.2±1.7	<.0001
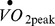 (L·min^−1^)	4.38±0.72	2.68±0.64	<.0001
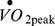 (ml·kg^−1^·min^−1^)	56.3±8.2	45.1±7.4	<.0001
Total METs	16.1±2.3	12.9±2.1	<.0001
Exercise Field Test			
Distance (m)	2700±400	2200±400	<.0001

Mean ± one standard deviation are reported.

*BMI* body mass index, *bpm* beats per minute, *METs* metabolic equivalents, 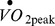 peak oxygen uptake.

### Protocol

Each subject completed an ETT and then an EFT on separate days with at least a 48 hour interval and no more than 2 weeks between sessions. The ETT was conducted first to allow investigators to continuously monitor the subject in a controlled environment and ensure that the subject could safely complete the subsequent EFT without any adverse events. Subjects were instructed to not vary their physical activity levels between sessions. Subjects were asked to refrain from eating or drinking food or caffeine within three hours of testing. Physical exercise and use of alcohol were prohibited on the day prior to and the day of testing. Prior to testing, age predicted maximum heart rate (HR_max_) was calculated for each subject [20]. Subjects' height and mass were measured to the nearest 0.5 cm and 0.1 kg, respectively, and body mass index (BMI) was calculated as mass (kg) divided by height (meters, [m]) squared. Before each session, subjects were fitted with a commercially available HRM (Polar Coded 31 Transmitter and OEM module, Polar Electro Oy, Kempele, Finland), and resting blood pressure and heart rate were assessed to screen for contraindications to ETT.

Participants performed a symptom-limited ETT by the Bruce protocol [21] on a motorized treadmill until volitional exhaustion. Symptoms, heart rate, and blood pressure (measured by arm-cuff sphygmomanometry) were recorded during the last minute of each exercise stage, at test termination (peak exercise), and 1 and 2 minutes into recovery, which comprised a controlled walk at 3.2 km per hour and a 0 percent grade. Ventilation and gas exchange were measured (TrueOne 2400 Metabolic Measurement System, Parvo Medics, Sandy, UT) and reported as the mean value per 15-second epoch. Criteria for maximal oxygen consumption rate (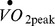) (i.e. respiratory exchange ratio > 1.1 and HR_max_ within 10 beats of age-predicted reference value) were achieved in most subjects; however, a plateau of oxygen consumption rate was not observed in all cases [22]. Therefore, the highest 15-second mean value of oxygen consumption (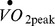) was determined and used in subsequent analyses. Respiratory exchange variables were used to estimate energy expenditure per minute and metabolic equivalents (METs) [23]. Heart rate was measured continuously by the HRM and as a mean value per 15-second epoch by a Polar receiver module connected to the metabolic cart. HR_max_ was defined as the heart rate value obtained at peak exercise. HRR at 1- and 2-minutes were defined as the reduction in heart rate from HR_max_ to 1 minute and 2 minutes after cessation of exercise, respectively.

Subjects completed an EFT outdoors on either a rubberized running track or hard dirt trail. They were instructed to run the maximum tolerated distance in 12 minutes. Most subjects completed the test while running; however, two subjects walked intermittently. Verbal cues of the remaining test time were called out after the first lap, halfway, and with one minute remaining. Upon test completion, total distance to the nearest 100 m mark was recorded, and the subject walked at a self-selected pace for at least 5 minutes in recovery. HRM data were used to determine HR_max_ and HRR values.

### Statistical Analysis

All statistical analyses were performed in SAS (version 9.3, SAS Institute, Cary, NC). Means and standard deviations were calculated for subject demographics and exercise parameters. Differences between sexes were assessed using t-tests with significance defined as p<0.05. A general linear model was fit by using the EFT data and the SAS software procedure GLM to predict 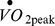. Hypothesized predictors of 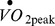 included distance, sex, HR_max_, age, and BMI. Backward selection was conducted with the goal of minimizing the prediction error as measured by the predicted residual sum of squares (PRESS) statistic. Significance was defined at p<0.05. The model was validated using leave-one-out cross validation, and the PRESS statistic was calculated to compare prediction accuracy between models. As Cooper's model was not derived from our sample, two approaches were used to obtain an estimate of the prediction error. First, the sum of squared prediction errors was calculated for Cooper's model. Second, we re-fit Cooper's model to our sample (i.e. only distance was used to predict relative 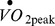) to obtain a PRESS estimate. The PRESS statistic or sum of square prediction errors, root mean square error (RMSE), and Pearson correlation coefficients for the new model, the re-fit model, and Cooper's model were compared. Predictions from the new model were compared to those produced using Cooper's model to assess bias (under- or over-estimation), prediction error (observed – predicted), and the associated standard deviation. The degree of agreement between HR_max_ and HRR values obtained by (1) the metabolic cart and HRM during the ETT and (2) the HRM during the ETT and EFT were assessed. The difference in means was used to estimate bias, and Pearson correlation coefficients were calculated as a measure of relative reliability. To obtain objective measures of agreement, equivalency tests were conducted using the SAS software procedure TTEST with the two one-sided tests (TOST) option with upper and lower bounds specified at ±6 beats per minute (bpm). These equivalency limits were based on a previous study reporting that heart rates measured by a Polar HRM and electrocardiogram equipment were within 6 bpm [24]. The validity of the normality assumption was assessed using histograms and QQ plots of the residuals.

## Results

The general physical and physiological characteristics of the subjects tested were unremarkable. There were significant differences between men and women in height (p<.0001), body mass (p<.0001), BMI (p = 0.0008), and resting systolic blood pressure (p = 0.0020) (Table 1). There was no difference in age (p = 0.33) between the sexes. Men achieved greater performance than women during the ETT and EFT as indicated by the exercise time (p<.0001), 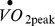 (p<.0001), total METs achieved (p<.0001), and distance completed (p<.0001).

### Prediction of 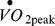 from a 12-Minute Run/Walk Exercise Field Test

Our final model that minimized the prediction error of 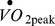 from a 12-minute run/walk included distance (p<.0001) and sex (p = 0.0281) and had lower prediction errors than the re-fit Cooper model and the Cooper model (Table 2). There was insufficient evidence that HR_max_ (p = 0.96), age (p = 0.42), and BMI (p = 0.30) improved the performance of our model. Our model (expressed by two equations, one for men and one for women), the re-fit Cooper model, and the original Cooper model are reported in Equations 1-4 and Table 3).

**Table 2:** PRESS statistic, RMSE, and correlation coefficients for our model, the re-fit Cooper model, and the Cooper model.

	PRESS	RMSE (ml·kg^−1^·min^−1^)	Correlation (r)
Our Model	595.6	3.45	0.88
Re-fit Cooper Model	636.4	3.57	0.87
Cooper Model	3498*	8.36	0.90

The correlation coefficient reported for the Cooper model was based on the original study [7].

*PRESS* predicted residual sum of squares, *RMSE* root mean square prediction error.

* Sum of squared prediction errors was used rather than PRESS for Cooper's model as the error was not based on a fitted model.

**Table 3:** Standard errors and p values for our model and the re-fit Cooper model developed to predict relative 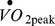 (ml·kg^−1^·min^−1^) from a 12-minute run/walk EFT.

	Our Model		Re-fit Cooper Model	
	SE	p	SE	p
Intercept	2.788	= .0795	2.707	= .323
Distance	1.981	<.001	1.753	<.001
Sex (male/female)	1.110	= .0281	-	-

*SE* standard error.

Our model for men (Equation 1):
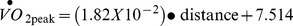



Our model for women (Equation 2): 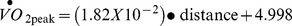



Re-fit Cooper model (Equation 3): 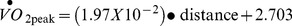



Original Cooper model (9) (Equation 4): 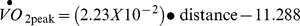




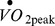
 and distance are expressed in ml·kg^−1^·min^−1^ and meters respectively.

**Figure 1:**
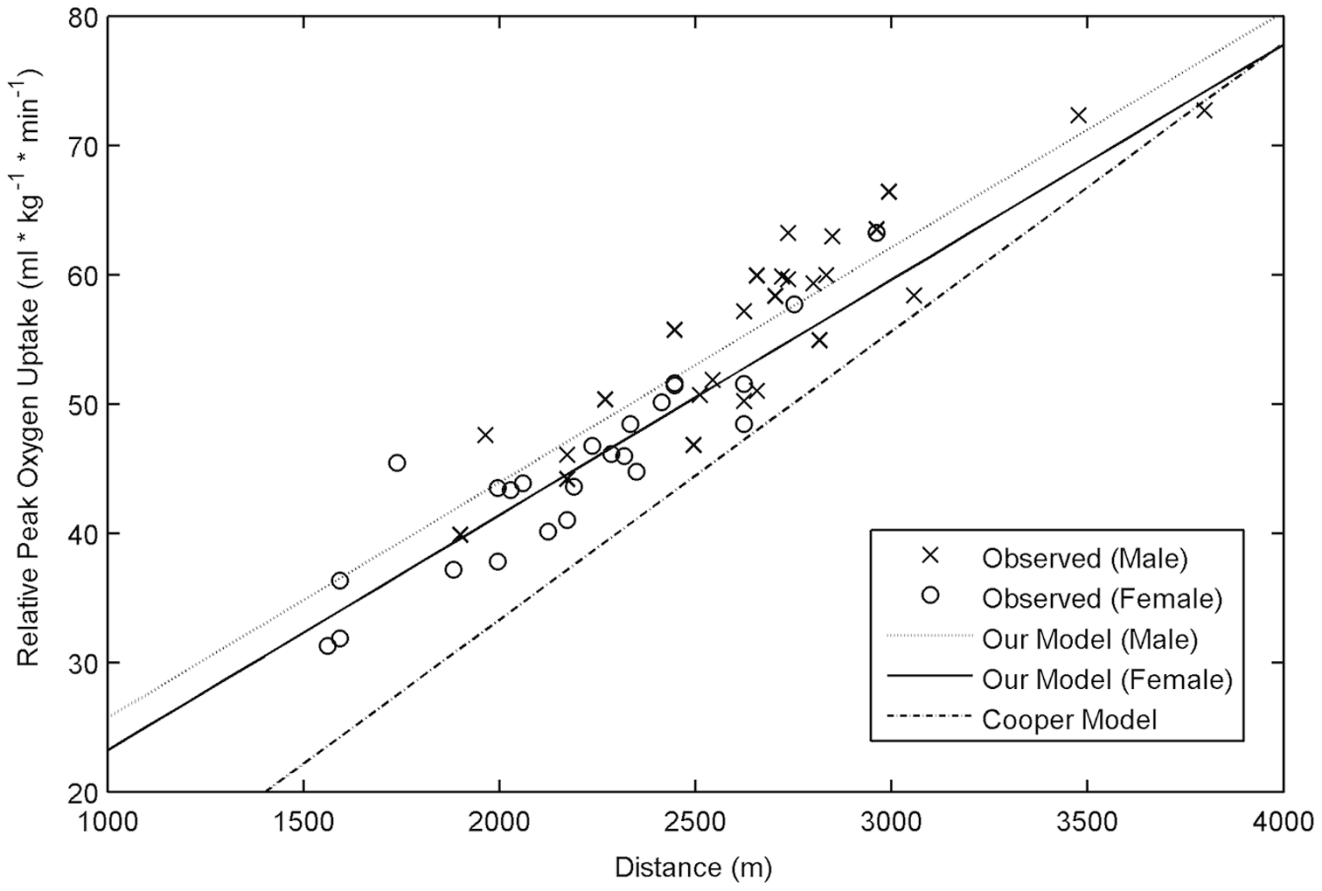
Comparison of observed 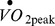 values to our model and the Cooper model. Our model was plotted for males and females. The Cooper model underestimated observed 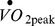 in all but one male subject and demonstrated increased error with decreased 12-minute run/walk distance.

Predicted 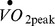
values determined from our model and the Cooper model were compared with observed 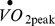 values (Figure 1). The Cooper model demonstrated a consistent downward bias and underestimated 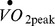 by an average of 7.55±3.64 ml·kg^−1^·min^−1^. The underestimation of 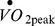 was most pronounced at the lower levels of CRF (Figure 1). The average absolute differences between observed and predicted 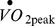 values using our model indicate a slight underestimation of 0.26±3.27 ml·kg^−1^·min^−1^.

### Comparison of Heart Rate Recovery between an Exercise Tolerance Test and a 12-minute Run/Walk Exercise Field Test

HR_max_ and 1- and 2-minute HRR measured by the metabolic cart demonstrated a strong relationship and were equivalent within 6 bpm of the same values measured by the HRM (Table 4). HR_max_ at the time of the ETT and EFT both measured by the HRM were equivalent (Table 5). HRR at 1- and 2-minutes were outside the specified equivalency limits; however, correlation coefficients demonstrate a moderate relationship between the ETT and EFT values (Table 5).

**Table 4:** Heart rate parameters obtained by the metabolic cart and HRM during the ETT (means ± SD), correlation between metabolic cart and HRM, bias, and two one-sided test (TOST) equivalency limits.

Parameter	Metabolic Cart	HRM	Correlation (r)	Bias	TOST 95% Equivalency Limits	Equivalent
HR_max_ (bpm)	187±8.6	190±8.9	0.99 (p<.001)	−2.50	−2.17 to −2.82	Yes
HRR1 (bpm)	23±8.4	27±9.4	0.92 (p<.001)	−4.34	−3.49 to −5.19	Yes
HRR2 (bpm)	44±13.1	48±12.5	0.97 (p<.001)	−3.86	−3.06 to −4.66	Yes

Upper and lower equivalency bounds were defined as 6 bpm.

Bias: Difference between metabolic cart and HRM parameters.

*BPM* beats per minute, *HRM* heart rate monitor, *HR_max_* maximum heart rate, *HRR1* heart rate recovery at 1 minute post-exercise, *HRR2* heart rate recovery at 2 minutes post-exercise, *TOST* two one-sided test.

**Table 5:** Heart rate parameters obtained by the HRM during the ETT and EFT (means ± SD), correlation between ETT and EFT, bias, and two one-sided test (TOST) equivalency limits.

Parameter	ETT	EFT	Correlation (r)	Bias	TOST 95% Equivalency Limits	Equivalent
HR_max_ (bpm)	190±8.9	193±9.2	0.74 (p<.001)	−3.13	−4.68 to − 1.59	Yes
HRR1 (bpm)	27±9.4	33±10.1	0.75 (p<.001)	−6.12	−7.75 to −4.49	No
HRR2 (bpm)	48±12.5	52±12.7	0.68 (p<.001)	−3.90	−6.31 to −1.49	No

Upper and lower equivalency bounds were defined as 6 bpm.

Bias: Difference between ETT and EFT parameters.

*BPM* beats per minute, *EFT* exercise field test, *ETT* exercise treadmill test, *HR_max_* maximum heart rate, *HRR1* heart rate recovery at 1 minute post-exercise, *HRR2* heart rate recovery at 2 minutes post-exercise, *TOST* two one-sided test.

## Discussion

Traditional methods for risk stratification for cardiovascular death are improved with inclusion of measures of both CRF and HRR [4], thus, low-cost methods to assess CRF and HRR in large populations outside of clinical settings may enable early risk detection for cardiovascular disease and identify asymptomatic individuals in need of additional clinical risk evaluations. In this study, we developed a CRF (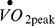) prediction model applicable to both men and women with a greater fitness range than the Cooper model [9] and evaluated the accuracy of HRR during a 12-minute run/walk EFT in an asymptomatic, low-risk population. Our linear model includes sex and distance achieved during a 12-minute run/walk EFT to predict 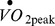. Our model improved accuracy for women and subjects with low CRF compared to the Cooper model (Figure 1) suggesting that it may be a useful method for screening for cardiovascular disease risk. We also demonstrated that a HRM is a valid tool to assess HRR. Lastly, we found that there was insufficient agreement of HRR values for ETT and EFT obtained by a HRM for interchangeable use. These differences, however, may be attributable to variations in test conditions and day-to-day variations in HRR and highlight a need to determine the effect of this variability on HRR prognostic criteria.

### Prediction of 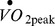 from a 12-Minute Run/Walk Exercise Field Test

The improved predictive accuracy of our CRF model over the Cooper model [9] is likely due to differences in the populations studied. While the mean of 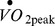 for our study population was above average (> 90^th^ percentile based on the average age) [25], there was sufficient variability in fitness to expand the range of 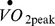 (31–72.7 ml·kg^−1^·min^−1^) and 12-minute run/walk distances (1561–3798 m) compared to the Cooper study [9]. As our population included not only a wider range of fitness levels, but also both sexes, it is not surprising that Cooper's model underestimated 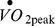 values in our population, a finding consistent with previous studies [11], [14], [15]. CRF is known to be greater in men than women due to differences in cardiac output and arterial-venous oxygen difference [26], [27]. Therefore, the inclusion of sex as an independent predictor of 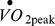 is an important improvement to the CRF prediction model to control for this source of variability. The clinical utility of an EFT is the identification of patients with low CRF and autonomic dysfunction as evidenced by low HRR, a population at increased cardiovascular risk [4]. Given that our model differentiates between sex and improved predictive accuracy in women and those with low CRF, it has the potential to be a useful tool for screening of pre-clinical cardiovascular disease.

### Comparison of Heart Rate Recovery between an Exercise Tolerance Test and a 12-minute Run/Walk Exercise Field Test

HRR values for ETT and EFT obtained by a HRM had insufficient agreement for interchangeable use. Preference for a slower recovery speed following the EFT may have increased HRR values in some subjects. HRR cut-off values have been demonstrated to vary by 6 bpm between active and passive recoveries [28]. Standardization of recovery in the resting supine position in our protocol, therefore, could have improved the equivalency of HRR values between the ETT and EFT. Further, variation in HRR between successive symptom-limited exercise tests is not well established, and the equivalency bounds may have been too restrictive. This concept is supported by a previous HRR reliability study that reported standard errors of approximately 10 bpm for two maximal exercise tests completed within 72 hours [29]. Variation in day-to-day HRR may result from external factors known to affect heart rate (e.g. time of day, ambient temperature, mental stress, and hydration status) [30], but it is difficult to minimize the effect of these variables during exercise testing.

The ease of administering an EFT with a commercial heart rate monitor may improve accuracy and precision of HRR measurement to alleviate some of these issues. Imai and colleagues [31] demonstrated that parasympathetic reactivation is greatest in the first 30 seconds of recovery with rapid declines observed in athletes. The continuous measurement ability of the HRM can assess this rapid change and could increase the sensitivity of HRR calculations compared to clinical systems that use data averaging or smoothing algorithms. Further, the low-cost nature of an EFT permits the test to be performed serially. An average of multiple HRR values could mitigate the effect of day-to-day variations on HRR prognostic reliability.

### Limitations

Testing a population with greater diversity than tested here and in previous studies (e.g. age, BMI, health status) is a logical next step to refine and increase the applicability of an EFT CRF model and to assess the prognostic value of EFT HRR. The population tested in this study was more diverse than Cooper's but otherwise had little variance in age and BMI, or other variables that are known to affect CRF [32], [33] and are significant in prediction models developed for shorter field tests [16], [17]. Expansion of this study's testing protocol to a more diverse population is necessary to adequately distinguish the influence of these variables on 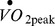 in the EFT. It should also be appreciated that a 12-minute run/walk EFT is not appropriate for all individuals. We believe that persons with functional limitations that impair their ability to walk or run, or with poor exercise capacity, or symptoms of cardiac ischemia would be poor candidates for an EFT. Such patients could be identified by a tool such as the Duke Activity Status Index as is the case for pre-operative surgical evaluation [34], [35]. For people with sufficient exercise capacity, the EFT may provide a valuable screening alternative for some clinical populations due to the ability of our model to predict exercise capacity (i.e. 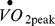) better than previous models in subjects with low CRF. HRR assessments were made using intra-individual comparisons thereby reducing population effects; however, selection of a healthy population rather than patients referred for clinical indications limited assessment of the prognostic value of HRR.

## Conclusions

A 12-minute run/walk EFT completed while wearing a HRM was demonstrated as an effective method to estimate CRF and measure HRR. Linear regression models for men and women were developed to predict 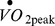 from 12-minute run/walk distance in an asymptomatic population between 18–45 years of age. These models are more accurate than the commonly used Cooper model. In addition, HRMs capable of continuously measuring heart rate were shown to accurately assess HRR compared to clinical equipment. Use of this technology with the completion of multiple EFTs could improve the accuracy and precision of HRR measurements. Collectively, these findings indicate that field-based evaluations of CRF and HRR are feasible and warrant further investigation as an inexpensive approach to screen and monitor cardiovascular disease risk in large asymptomatic populations.
